# Korea Youth Risk Behavior Survey: achievements in the last 20 years and future directions

**DOI:** 10.4178/epih.e2026025

**Published:** 2026-06-08

**Authors:** Yangha Kim, Yunjung Choi, Suyeon Park, Ji-Su Park, Sun-Ja Kim, Yoonjung Kim, Sungha Yun, Sunhye Choi, Soyeon Kim, Kyungwon Oh

**Affiliations:** Division of Health and Nutrition Survey and Analysis, Bureau of Chronic Disease Prevention and Control, Korea Disease Control and Prevention Agency, Cheongju, Korea

**Keywords:** Korea Youth Risk Behavior Survey, Adolescents, Health behavior, Health policy

## Abstract

The Korea Youth Risk Behavior Survey (KYRBS) has been conducted annually by the Korea Disease Control and Prevention Agency (KDCA) since 2005 to monitor trends in health behaviors among Korean adolescents, with the 20th survey completed in 2024. Each year, approximately 60,000 middle and high school students participate in an anonymous, self-administered, web-based survey comprising approximately 110 health behavior items. Over the past 20 years, school-level and student-level participation rates averaged 99.8% and 94.9%, respectively, with a cumulative total of 1,304,561 participants. The prevalence of current cigarette smoking decreased from 11.8% (male, 14.3%; female, 8.9%) in 2005 to 3.6% (male, 4.8%; female, 2.4%) in 2024, and the prevalence of current drinking decreased from 27.0% (male, 27.0%; female, 26.9%) to 9.7% (male, 11.8%; female, 7.5%). In contrast, the prevalence of skipping breakfast increased from 27.1% (male, 26.4%; female, 28.0%) to 42.4% (male, 40.2%; female, 44.7%), whereas the prevalence of depressive symptoms showed little overall change (29.9% [male, 25.6%; female, 34.7%] in 2005 and 27.7% [male, 23.1%; female, 32.5%] in 2024). Survey data are released within the same year of collection and have been used as key evidence for the development and evaluation of adolescent health policies, including the Master Plans for Student Health Promotion and the National Health Plan in Korea. The KYRBS will continue to develop new survey items reflecting emerging health issues, expand the target population to include children, and regularly produce regional-level adolescent health statistics, thereby maintaining its role as a key national health surveillance system.

## INTRODUCTION

Adolescence is a critical developmental period during which lifelong health behaviors are established and may directly influence the risk of chronic diseases in adulthood [1]. In response to this need, the World Health Organization (WHO) initiated the Health Behaviour in School-aged Children survey in 1983 and the Global School-based Student Health Survey in 2003, while the United States introduced the Youth Risk Behavior Surveillance System in 1991 [2-4].

Recognizing the need for national-level monitoring of adolescent health behaviors, the Korea Disease Control and Prevention Agency (KDCA) planned the Korea Youth Risk Behavior Survey (KYRBS) in 2004 as part of the National Chronic Disease Surveillance System. The survey was launched in 2005 and has since been conducted annually [5], with the 20th survey completed in 2024.

As of 2024, the KYRBS targets approximately 60,000 students from 800 sample schools selected nationwide through a stratified cluster sampling design. The survey includes approximately 110 items across 15 domains, including tobacco use, alcohol use, physical activity, dietary behaviors, obesity, and mental health. It is conducted as an anonymous, self-administered online survey using mobile devices during regular class hours from June to August. Survey results are released in December of the survey year, and raw data are publicly available through the KYRBS website (https://kdca.go.kr/yhs/).

This paper reviews the major achievements of the KYRBS over the past 20 years and outlines its future directions.

## MAJOR ACHIEVEMENTS OVER THE PAST 20 YEARS

A national surveillance system capable of continuously monitoring health behaviors among Korean adolescents has been established, enabling systematic assessment of changes over the past 2 decades. The accumulated data have served as key evidence for the development and evaluation of adolescent health policies. In addition, publicly available data have been widely used across multiple academic disciplines, thereby contributing to the promotion of adolescent health.

### Establishment of the adolescent health surveillance system

#### Sustainability of the survey system

Since its establishment by the KDCA in 2005, the school-based KYRBS has been conducted continuously and reached its 20th cycle in 2024. From the planning stage, a joint survey system was developed in collaboration with the Ministry of Education to ensure that survey findings could be effectively linked to policy development. To maintain a stable operational framework, the KYRBS Steering Committee—comprising officials from the Ministry of Education and 17 metropolitan and provincial offices of education—has convened annually to strengthen cooperation with educational authorities and support effective survey implementation. As a result, average participation rates over the past 20 years remained high, reaching 99.8% at the school level and 94.9% at the student level (Table 1), which was approximately 24 percentage points higher than the student participation rate reported for the Youth Risk Behavior Surveillance System (71.0% in 2023) [6].

In addition, an advisory committee reviews survey items and findings across domains, as well as issues related to data release, thereby helping to ensure survey quality and data reliability.

#### Ensuring representativeness

To reflect updated information on city size, school and student populations, and types of high schools, the sampling frame is updated annually using the most recent Statistical Yearbook of Education (April 2023 data for the 2024 survey). Using student population data from the year preceding the survey, a 2-stage stratified cluster sampling design is applied, with schools selected in the first stage and classes selected in the second stage. A total of 800 schools are selected using a permanent random number sampling method, and 1 class per grade is randomly selected within each school, resulting in 2,400 sample classes. The target population includes approximately 60,000 students attending 800 sample schools nationwide, including 400 middle schools and 400 high schools. When the survey was first implemented in 2005, it was conducted in September and excluded third-year high school students because of its proximity to the College Scholastic Ability Test; however, third-year high school students have been included since 2006.

This sampling approach has been maintained since the early years of the survey. As of 2024, 799 of 5,673 schools (14.1%) and 58,285 of 2,612,077 students (2.2%) were sampled. The sample therefore covers 14.1% of all schools and is considered sufficiently representative for a national-level survey.

#### Gradual expansion of survey items

When the KYRBS was first implemented in 2005, it consisted of 92 items across 11 domains (Table 2), including tobacco use, alcohol use, physical activity, dietary behaviors, and mental health. In response to policy and research demands, additional domains were sequentially introduced, including health equity (2006), atopic dermatitis and asthma (2007), internet addiction (2008), and violence (2012), resulting in a total of 106 items across 15 domains by 2024.

Survey items were structured to reflect social and environmental changes affecting adolescent health. For example, following the domestic introduction of heated tobacco products in 2017, tobacco-related items were expanded in 2019 to distinguish among cigarettes, electronic vapor products, and heated tobacco products, thereby enabling assessment of product-specific use and multiple-product use. In the physical activity domain, given that physical activity levels among Korean adolescents are substantially lower than those reported in other countries [7,8], items addressing barriers to physical activity (2016) and facilitators of physical activity (2022) were added. In the dietary behavior domain, reflecting changes in beverage consumption patterns and the food environment, existing items on carbonated beverage and energy drink consumption were revised in 2022 to assess sugar-sweetened beverage and high-caffeine beverage consumption, and an item on zero-calorie beverage consumption was newly introduced in 2025. Furthermore, in response to concerns regarding deterioration in adolescent mental health during the coronavirus disease 2019 (COVID-19) pandemic, new items assessing anxiety and loneliness were added in 2020, followed by items on smartphone use in 2021 and post–COVID-19 symptoms in 2022.

**Figure 1.**
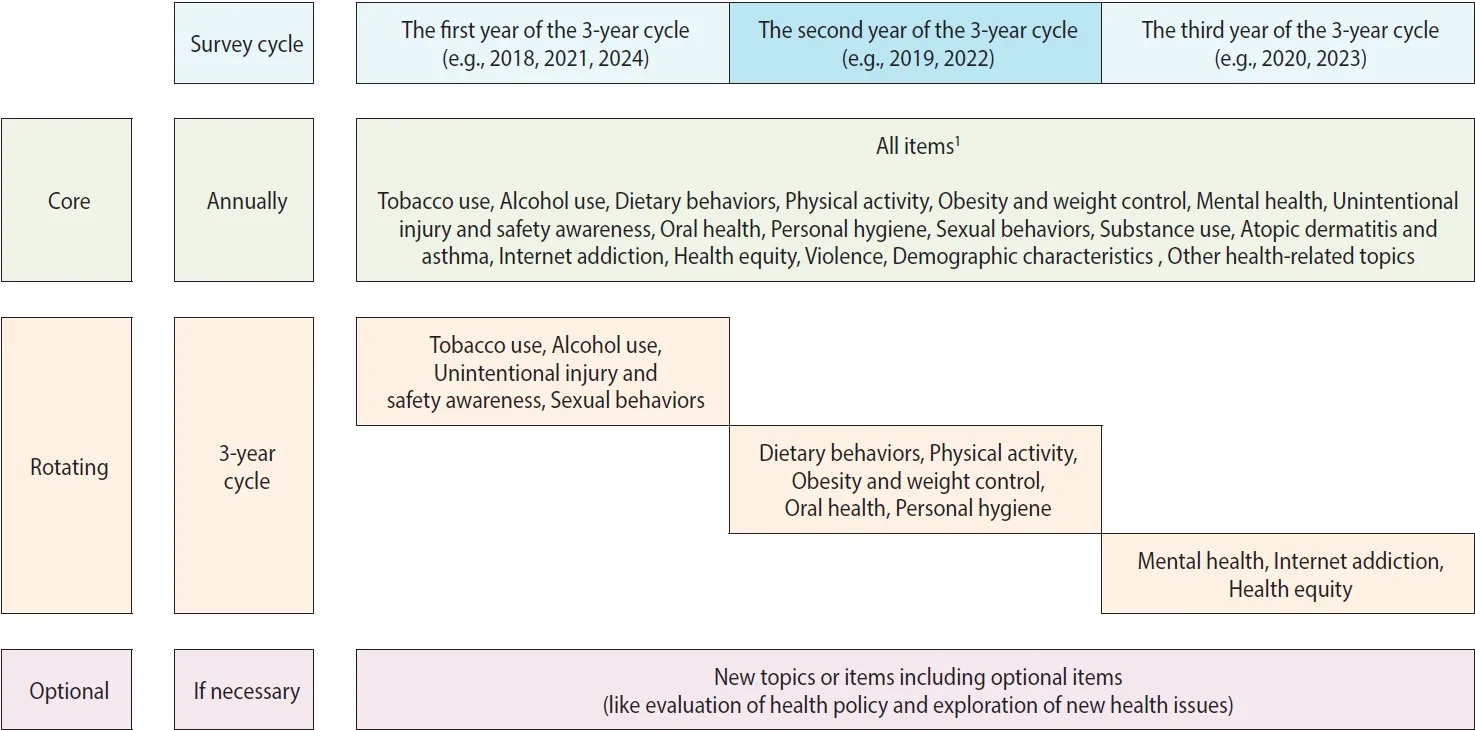
Rotational survey system of the Korea Youth Risk Behavior Survey. ^1^Rotating domains, introduced in 2018, are assessed according to a 3-year cycle consisting of the first, second, and third years of each cycle.

Although newly proposed items reflecting changes in adolescent health behaviors were actively incorporated, maintaining a manageable number of items remained necessary to ensure survey completion within a limited time frame. Accordingly, since 2018, a rotational survey system has been implemented in which all survey items are classified as core, rotating, or optional according to policy relevance and year-to-year variability. Core items (e.g., current tobacco or alcohol use) are surveyed annually and include indicators with high policy utility, such as those used to evaluate national health policies (e.g., the National Health Plan), indicators used for international comparisons, or items showing substantial temporal variability. Rotating items include factors related to changes in health behaviors (e.g., parental or close friends’ smoking status) or indicators with relatively low variability (e.g., reasons for skipping breakfast) and are surveyed on a 3-year cycle. Optional items are introduced as needed in response to policy changes (e.g., changes in smoking behavior following the 2015 tobacco price increase) or major social events (e.g., post–COVID-19 symptoms in 2022) (Figure 1).

#### Improving convenience of participation

At its introduction, the KYRBS was administered as an anonymous, self-administered online survey using desktop computers during class hours. Since 2020, with the expansion of wireless internet infrastructure in schools following the introduction of mandatory software education and remote classes during the COVID-19 pandemic, educational digital devices have been gradually distributed [9]. In addition, given the high smartphone ownership rate among Korean adolescents (99.6%) [10], the feasibility of introducing a mobile-based survey was assessed to improve participation convenience.

In 2022, a qualitative study and pilot survey were conducted to assess the feasibility of transitioning to mobile devices [11,12]. The qualitative study included 21 students from the second year of middle school to the second year of high school and used focus group interviews. Smartphones were perceived as more convenient for selecting responses and entering open-ended answers, and students reported greater confidence in anonymity than when using desktop computers. However, survey responses did not differ by mode of survey administration. The pilot survey was conducted in a subset of sample schools participating in the 2022 KYRBS (22 schools, 132 classes, 2,754 students), in which additional classes completed the survey using smartphones. Except for a few indicators, such as current heated tobacco product use and depressive symptoms, no significant differences were observed in major health indicators, including current cigarette smoking, current drinking, and physical activity for at least 60 min/day on ≥5 day/wk.

Based on these findings, beginning in 2023, the mode of survey administration was changed to allow participation using mobile devices, including tablets or smartphones, during class hours (Table 2). In addition, no significant differences were observed in major health indicators between mobile device types [13], allowing schools to select the survey device according to their circumstances. This transition improved convenience for students and reduced the administrative burden on teachers and schools.

#### Enhancing timeliness of data release

From 2005 to 2011, the survey was conducted between September and November, and the results and raw data were released in June of the following year. To support planning of health promotion policies for the subsequent year based on current findings, the survey period was shifted to June–August beginning in 2012, and survey results began to be released in December of the same year. Raw data have also been publicly released through the KYRBS website (https://kdca.go.kr/yhs/), ensuring timely dissemination. However, during the COVID-19 pandemic (2020–2023), the survey period was temporarily moved back to September–November.

### Monitoring changes in adolescent health behaviors

#### Tobacco use and alcohol use

The prevalence of current cigarette smoking in 2024 was 4.8% among male students and 2.4% among female students, representing an approximately threefold decrease over the past 20 years. In contrast, the prevalence of electronic vapor product or heated tobacco product use in 2024 was 4.4% among male students and 2.4% among female students, with an increasing trend observed among female students compared with 2019 (Table 3).

The prevalence of current tobacco product use in 2024 was 5.8% among male students and 3.2% among female students. Compared with 2019, this prevalence decreased among male students but showed no significant change among female students (Table 3). Compared with United States high school students, the prevalence of current cigarette smoking was 2.1 percentage points higher among Korean high school students (Korea, 5.6%; United States, 3.5%), whereas the prevalence of current tobacco product use was 10.3 percentage points lower (Korea, 6.8%; United States, 17.1%) [8].

Among current tobacco product users, more than 60% reported using 2 or more types of tobacco products, including cigarettes, electronic vapor products, and heated tobacco products (2024: male students, 62.1%; female students, 60.6%). Multiple-product use increased in both sexes, with a particularly large increase among female middle school students (data not shown).

The prevalence of current drinking in 2024 was 11.8% among male students and 7.5% among female students, showing a decline of similar magnitude to that observed for current cigarette smoking compared with 2005. By school level, high school students consistently had a higher prevalence than middle school students, whereas larger reductions over the past 20 years were observed among middle school students of both sexes. The prevalence of risky drinking also declined substantially over the same period (2024: male students, 4.8%; female students, 3.8%), with school-level patterns similar to those observed for current drinking (Table 3).

#### Physical activity and dietary behaviors

The prevalence of physical activity, defined as engaging in physical activity for at least 60 min/day on ≥5 day/wk, was 25.1% among male students and 8.9% among female students in 2024, representing an increase compared with 2009. However, compared with United States high school students (46.3% in 2023), the prevalence among Korean high school students remained low (12.9% in 2024) [8], indicating the need for further improvement. The prevalence of vigorous-intensity physical activity, defined as engaging in vigorous-intensity physical activity on at least 3 day/wk, was 53.5% among male students and 28.8% among female students in 2024, representing an increase compared with 2022; the largest increase was observed among male middle school students (7.3 percentage points) (Table 4).

The prevalence of skipping breakfast on at least 5 day/wk was 40.2% among male students and 44.7% among female students in 2024, showing an increasing trend since 2005.

The prevalence of fruit consumption at least once per day was 18.3% among male students and 19.0% among female students in 2024. The prevalence decreased in both sexes after 2005, with a more pronounced decline among female students after 2011 (Table 4). In addition, the prevalence of consuming energy drinks and high-caffeine beverages at least 3 times/wk increased, reaching 23.2% among male students and 23.9% among female students in 2024 (data not shown).

#### Mental health

The prevalence of depressive symptoms in 2024 was 23.1% among male students and 32.5% among female students, representing a decrease in both sexes compared with 2005. However, this trend was not statistically significant after 2016. The prevalence of generalized anxiety disorder (10.3% among male students and 18.0% among female students in 2024) and loneliness (14.3% among male students and 23.6% among female students in 2024) increased in both sexes compared with 2020, with larger increases observed among middle school students (Table 5).

### Utilization in adolescent health policy

KYRBS data have been used throughout the policy cycle, including identifying adolescent health problems, establishing policies based on identified priorities, and monitoring and evaluating policy implementation and outcomes.

In particular, under the fifth National Health Promotion Plan (Health Plan 2030, 2021–2030; Ministry of Health and Welfare), 17 performance indicators across 3 domains—tobacco control, alcohol control, and physical activity—have been monitored using KYRBS data since 2021 to assess progress toward policy targets. In addition, KYRBS indicators on smoking, alcohol use, physical activity, dietary behaviors, and oral health have been used to develop national plans, set targets, and monitor target achievement. These plans include the First (2019–2023) and Second (2024–2028) Master Plans for Student Health Promotion (Ministry of Education), the Second (2017–2021) and Third National Nutrition Management Plans (Ministry of Health and Welfare), and the First and Second Oral Health Plans (Ministry of Health and Welfare).

Across health domains, KYRBS indicators have also informed and supported evaluation of policy initiatives. In the tobacco domain, KYRBS data have been used to designate and expand school smoke-free zones (1999–2024) and to evaluate the implementation outcomes of school-based smoking prevention and cessation programs. They have also served as key evidence supporting strengthened tobacco control policies, including the tobacco price increase from 2,500 Korean won (KRW) to 4,500 KRW in 2015 and the introduction of pictorial health warnings in 2016. These policy efforts contributed to a reduction in the prevalence of current cigarette smoking among adolescents, from 11.8% in 2005 to 3.6% in 2024.

In the physical activity domain, policies to promote adolescent physical activity, such as expanding school-based physical activity programs, have been implemented in response to the low prevalence of aerobic and muscle-strengthening activities. These efforts have been conducted under the Third Basic Plan for the Promotion of School Physical Education (2024–2028; Ministry of Culture, Sports and Tourism and Ministry of Education) and the Master Plans for Student Health Promotion. Although physical activity levels among Korean adolescents remain lower than those in other countries [7,8], they have shown gradual improvement, suggesting the potential effectiveness of these initiatives.

In the dietary behavior domain, findings such as high rates of skipping breakfast and frequent consumption of carbonated beverages and fast food have informed the Comprehensive Plan for the Safety Management of Children’s Dietary Life (since 2010; Ministry of Food and Drug Safety), as well as policies restricting the sale of high-calorie, low-nutrient foods and high-caffeine products in schools. However, some dietary indicators, including skipping breakfast and unhealthy food consumption, have not shown clear improvement, indicating the need for additional support and complementary measures.

Furthermore, based on high levels of perceived stress and depressive symptoms among adolescents, policies to strengthen protection and support for students vulnerable to mental health problems have been implemented within the Master Plans for Student Health Promotion. KYRBS data have also been used as baseline evidence for establishing directions for adolescent health promotion within the Community-based Integrated Health Promotion Program.

In addition, KYRBS data have been used to produce indicators for international monitoring, including insufficient physical activity under the WHO Global Action Plan for the Prevention and Control of Non-communicable Diseases and adolescent tobacco use and exposure to secondhand smoke at home under the Framework Convention on Tobacco Control. These indicators enable cross-national comparisons of adolescent health status.

### Enhancing the utilization of the data

To enhance the policy and academic use of the KYRBS, survey results and raw datasets have been actively disclosed to the public.

Each year, key findings are promptly disseminated to the public through press releases and results briefings during the year of data collection, and the raw datasets are made publicly available. As of August 2025, approximately 13,000 researchers had downloaded the raw data, and approximately 400 related research articles had been published, based on records from the KYRBS website and PubMed-indexed publications after excluding duplicates, theses, and conference abstracts. These studies have primarily examined the status of adolescent health behaviors and their associations with health outcomes, consistently reporting close associations between health behaviors and health status and emphasizing the need for active interventions to improve adolescent health behaviors [14-19].

In addition, fact sheets [20], issue briefs [13], and in-depth reports [21,22] have been published to stimulate public interest in adolescent health and health behaviors and to raise awareness of key health issues, thereby contributing to the development of youth health policies.

## FUTURE DIRECTIONS

Future directions for the KYRBS are organized around 3 areas: (1) improving the current survey system, (2) expanding the target population, and (3) producing regional-level health statistics. Beyond monitoring adolescent health behaviors, the KYRBS aims to support the development of tailored health promotion strategies at the regional and school levels.

### Improving the current survey system

The number of students enrolled in middle and high schools has steadily declined, from 65,482 in 2005 (excluding third-year high school students) to 57,580 in 2024. Because this decrease was not large enough to affect the production of national-level and provincial-level statistics, the sample size was maintained to ensure consistency in the sampling rate. Ongoing monitoring of changes in the number and characteristics of schools and students is needed to determine whether future sample-size expansion is warranted.

As previously reported [23], self-reported data tend to underestimate the prevalence of obesity and smoking, indicating the need for periodic assessment of measurement accuracy. However, no validation studies have been conducted since the 2018 review, underscoring the need for updated research to reassess the accuracy of self-reported measures.

Finally, although the anonymous, web-based, self-administered survey facilitates accurate responses, reduces logical errors, and shortens completion time, it limits linkage with other datasets, such as student health examination results. To enhance data utilization, further discussion is needed regarding methods for collecting limited personal identifiers while maintaining response accuracy.

### Expanding the target population

The KYRBS is a school-based survey and therefore has limitations in comprehensively capturing health behaviors among out-of-school adolescents. However, this gap has been addressed through the national Survey on Out-of-School Youth, which has been conducted by the Ministry of Gender Equality and Family since 2015 and monitors both policy needs and health behaviors among out-of-school adolescents. Rather than expanding the survey population to include out-of-school adolescents, the KYRBS aims to extend the target population to younger age groups. Alcohol and tobacco use begin at a relatively early age, around 13 years (13.5 years for alcohol use and 13.0 years for tobacco use) [24], and health problems such as media addiction and skipping breakfast have also been reported among elementary school students [25,26]. Considering these findings, the KYRBS seeks to expand its target population to include elementary school students to enable proactive intervention for health risk behaviors.

### Producing regional-level health statistics

The KYRBS was originally designed to generate national-level and provincial-level statistics on adolescent health behaviors. However, as demand for city-level, county-level, and district-level (*si–gun–gu*) data has increased for regional health planning and adolescent health promotion programs, regional statistics have been supported on a voluntary basis since 2009.

Support includes technical assistance for sample design, use of the survey system, training for survey coordinators, and statistical estimation. To encourage broader regional participation, detailed information on available support and examples of data utilization will be shared at future Steering Committee meetings. In the long term, survey items reflecting region-specific health issues and characteristics will be added to provide statistics needed for adolescent health promotion at the local level.

## CONCLUSION

Over the past 20 years, the KYRBS has become a comprehensive national survey for monitoring changes in adolescent health behaviors in Korea. Through the gradual expansion of survey items, timely release of same-year statistics, and public access to raw data, the KYRBS has provided essential evidence for understanding trends in adolescent health. In particular, the survey has documented changes in key health indicators, including reductions in tobacco and alcohol use and improvements in physical activity, and has served as a key evidence base for setting priorities and evaluating adolescent health policies. Moving forward, by improving the current survey system, expanding the target population, and producing regional-level health statistics, the KYRBS aims to support early identification of health risk factors among children and adolescents and to continue its role as a key national surveillance system for evidence-based policy decision-making and health promotion.

### Ethics statement

This study used data from the Korea Youth Risk Behavior Survey (KYRBS), a nationally mandated government-administered public health surveillance system. Therefore, separate institutional review board approval was not required in accordance with national regulations.

## Figures and Tables

**Table 1.** Participation in the 2005–2024 Korea Youth Risk Behavior Survey

Variables	2005^1^	2006	2007	2008	2009	2010	2011	2012	2013	2014	2015	2016	2017	2018	2019	2020	2021	2022	2023	2024	2005–2024^2^
Total																					
Participation rate (%)	88.9	90.9	94.8	95.6	97.6	97.7	95.5	96.4	96.4	97.2	96.7	96.4	95.8	95.6	95.3	94.9	92.9	92.2	92.9	94.9	94.9
Target population (n)	65,482	78,593	78,834	78,713	76,937	74,980	79,202	76,980	75,149	74,167	70,362	67,983	64,991	62,823	60,100	57,925	59,066	56,213	56,935	57,580	1,373,015
Participants (n)	58,224	71,404	74,698	75,238	75,066	73,238	75,643	74,186	72,435	72,060	68,043	65,528	62,276	60,040	57,303	54,948	54,848	51,850	52,880	54,653	1,304,561
Middle school																					
Participation rate (%)	87.6	91.2	95.4	96.3	98.0	98.0	96.9	96.8	96.9	97.8	97.5	96.9	96.6	96.8	96.2	96.2	89.1	94.9	95.0	96.1	95.5
Target population (n)	40,559	41,015	40,673	40,433	39,207	38,323	39,704	38,541	37,680	36,975	35,193	33,251	31,985	31,242	30,551	30,100	27,885	29,535	29,884	30,257	702,993
Participants (n)	35,549	37,420	38,820	38,943	38,409	37,570	38,474	37,297	36,530	36,156	34,299	32,219	30,885	30,229	29,384	28,961	24,833	28,015	28,401	29,087	671,481
High school																					
Participation rate (%)	91.0	90.4	94.0	94.8	97.2	97.3	94.1	96.0	95.8	96.5	95.9	95.9	95.1	94.4	94.5	93.4	96.3	89.3	90.5	93.6	94.3
Target population (n)	24,923	37,578	38,161	38,280	37,730	36,657	39,498	38,439	37,469	37,192	35,169	34,732	33,006	31,581	29,549	27,825	31,181	26,678	27,051	27,323	670,022
Participants (n)	22,675	33,984	35,878	36,295	36,657	35,668	37,169	36,889	35,905	35,904	33,744	33,309	31,391	29,811	27,919	25,987	30,015	23,835	24,479	25,566	633,080

1 Third-year high school students were excluded in 2005 due to the College Scholastic Ability Test.

2 Average participation rate.

**Table 2.** Major changes in the Korea Youth Risk Behavior Survey over the past 20 years

Category	2005	2006	2012	2018	2023	2024
Population	From the first year of middle school to the second year of high school	From the first year of middle school to the third year of high school (2006~)				
Items	11 Domains, 92 items^1^	12 Domains, 112 items	15 Domains, 129 items	15 Domains, 103 items	15 Domains, 88 items	15 Domains, 106 items
	Tobacco use, alcohol use, dietary behaviors, physical activity, obesity and weight control, unintentional injury and safety awareness, sexual behaviors, mental health, oral health, substance use, personal hygiene^1^	2005 Survey items retained, with the addition of the health equity domain.	2006 Survey items retained, with the addition of the atopic dermatitis and asthma, internet addiction, and violence domains.		Same as above	
		-		Implementation of a rotating survey system (2018)		
Method	Anonymous, self-administered online survey					
Mode of administration	Desktop computers (personal computers) (2005–2022)				Mobile (tablets, smartphones) (2023~)	
Period	Sep-Nov (2005–2011)		Jun-Aug^2^ (2012~)			
Data release	Jun of the following year (2005–2011)		Dec of the survey year^2^ (2012~)			

1 Initial survey conducted in 2005.

2 From 2020 to 2023, the survey period and the timing of results release were temporarily modified due to the coronavirus disease 2019 (COVID-19) pandemic.

**Table 3.** Trends in tobacco and alcohol use in the Korea Youth Risk Behavior Survey, 2005–2024

Variables	2005	2006	2007	2008	2009	2010	2011	2012	2013	2014	2015	2016	2017	2018	2019	2020	2021	2022	2023	2024	Difference^1^	APC^2^	APC^2^ and year of any significant change in trend slope		
																					2005/2019 to 2024		Before	Change year	After
Current cigarette smoking^3^																									
Male	14.3	16.0	17.4	16.8	17.4	16.6	17.2	16.3	14.4	14.0	11.9	9.6	9.5	9.4	9.3	6.0	6.0	6.2	5.6	4.8	–9.5^***^	–6.4^***^	2.2	2011	–9.5^***^
Middle school	9.6	9.3	11.3	10.3	11.1	10.6	11.0	9.8	7.9	6.8	4.8	3.5	4.1	3.9	4.0	1.9	2.1	2.3	2.4	1.8	–7.8^***^	–9.6^***^	1.2	2011	–14.0^***^
High school	22.4	23.8	24.3	23.8	23.9	22.5	23.1	22.4	20.7	20.8	18.3	14.7	13.9	14.1	14.2	10.1	10.0	10.4	9.0	7.8	–14.6^***^	–5.7^***^	–0.3	2012	–8.3^***^
Female	8.9	9.2	8.8	8.2	7.6	7.1	6.5	5.9	4.6	4.0	3.2	2.7	3.1	3.7	3.8	2.7	2.9	2.7	2.7	2.4	–6.5^***^	–7.5^***^	–9.7^***^	2016	–3.8^***^
Middle school	6.3	5.9	6.6	5.4	5.1	5.1	4.8	4.3	2.8	2.3	1.7	1.3	1.8	2.1	2.3	1.5	1.6	1.8	1.9	1.6	–4.7^***^	–8.3^***^	–11.0^***^	2017	–1.2
High school	13.5	13.0	11.3	11.1	10.2	9.0	8.3	7.5	6.3	5.6	4.5	3.8	4.1	5.1	5.2	3.8	4.2	3.6	3.6	3.3	–10.2^***^	–7.4^***^	–9.8^***^	2015	–4.3^***^
Current use of electronic vapor products or heated tobacco products^4^																									
Male	-		-	-	-	-	-	-	-	-	-	-	-	-	5.9	3.1	4.2	5.3	4.5	4.4	–1.5^***^	–0.2	–12.1^***^	2021	9.2^***^
Middle school	-		-	-	-	-	-	-	-	-	-	-	-	-	2.6	1.1	1.7	2.1	2.2	1.9	–0.7	0.7	–9.3	2021	5.5
High school	-		-	-	-	-	-	-	-	-	-	-	-	-	8.9	5.1	6.8	8.6	7.0	6.8	–2.1^***^	–1.6	–9.8	2021	4.6
Female	-		-	-	-	-	-	-	-	-	-	-	-	-	1.8	1.2	2.0	2.4	2.6	2.4	0.6^***^	9.4^***^	14.7	2022	2.5
Middle school	-		-	-	-	-	-	-	-	-	-	-	-	-	1.0	0.7	1.2	1.7	2.0	1.6	0.6^***^	15.1^***^	28.4	2022	0.8
High school	-		-	-	-	-	-	-	-	-	-	-	-	-	2.5	1.7	3.0	3.2	3.2	3.3	0.8	11.7^***^	17.9	2022	3.0
Current tobacco product use^5^																									
Male	-		-	-	-	-	-	-	-	-	-	-	-	-	10.3	6.7	7.0	7.3	6.6	5.8	–4.5^***^	–7.9^***^	–14.4^***^	2021	–3.5^***^
Middle school	-		-	-	-	-	-	-	-	-	-	-	-	-	4.6	2.2	2.6	3.0	3.0	2.4	–2.2^***^	–7.4	–25.0^***^	2021	5.0
High school	-		-	-	-	-	-	-	-	-	-	-	-	-	15.4	11.0	11.4	12.0	10.4	9.2	–6.2^***^	–7.7^***^	–10.8^***^	2021	–5.8^***^
Female	-		-	-	-	-	-	-	-	-	-	-	-	-	4.1	2.9	3.3	3.4	3.5	3.2	–0.9^***^	–0.4	–4.9	2021	2.0
Middle school	-		-	-	-	-	-	-	-	-	-	-	-	-	2.5	1.7	1.9	2.3	2.7	2.1	–0.4	2.7	–2.5	2021	4.9
High school	-		-	-	-	-	-	-	-	-	-	-	-	-	5.6	4.1	4.7	4.6	4.4	4.3	–1.3	–3.8^***^	–8.2^***^	2021	–1.2^***^
Current drinking^6^																									
Male	27.0	30.5	29.6	26.1	23.7	23.5	23.7	22.7	19.4	20.5	20.0	17.2	18.2	18.7	16.9	12.1	12.4	15.0	13.0	11.8	–15.2^***^	–4.6^***^	0.6	2007	–4.8^***^
Middle school	17.5	18.6	18.7	15.4	14.2	14.1	12.8	11.3	9.5	9.9	8.8	7.5	8.5	9.5	8.0	5.8	6.2	7.7	6.6	5.8	–11.7^***^	–6.3^***^	–7.7^***^	2015	–4.0^***^
High school	43.4	44.4	41.9	37.5	33.5	32.9	34.2	33.4	28.8	30.5	30.0	25.3	26.2	26.6	25.0	18.3	18.8	22.9	19.7	17.9	–25.5^***^	–4.5^***^	–4.3^***^	2018	–5.1^***^
Female	26.9	26.5	25.7	22.6	18.2	18.3	17.1	15.8	12.8	12.6	13.1	12.5	13.7	14.9	13.0	9.1	8.9	10.9	9.0	7.5	–19.4^***^	–6.0^***^	–8.1^***^	2013	–4.4^***^
Middle school	18.9	17.7	18.3	14.7	12.3	12.4	11.1	9.2	7.0	6.6	5.9	5.5	6.7	7.4	7.1	5.0	5.0	6.3	5.1	4.1	–14.8^***^	–7.8^***^	–10.9^***^	2015	–2.4
High school	40.7	36.8	34.0	31.0	24.2	24.3	23.2	22.3	18.4	18.2	19.4	18.3	19.5	21.5	18.4	13.2	13.0	15.9	13.2	11.0	–29.7^***^	–5.5^***^	–10.5^***^	2010	–4.2^***^
Risky drinking^7^																									
Male	10.5	13.0	12.5	10.7	10.6	10.4	11.0	10.3	8.7	9.5	9.6	8.5	8.8	9.1	8.2	5.6	5.3	6.1	5.4	4.8	–5.7^***^	–4.3^***^	–2.7^***^	2018	–9.6^***^
Middle school	3.7	3.9	3.9	3.1	3.4	3.4	3.3	2.8	2.3	2.2	2.1	1.7	2.2	2.6	2.1	1.4	1.5	1.7	1.6	1.3	–2.4^***^	–5.4^***^	–4.0	2010	–5.7^***^
High school	22.1	23.7	22.1	18.9	17.9	17.5	18.4	17.4	14.8	16.3	16.4	14.0	14.3	14.8	13.6	9.7	9.2	10.7	9.2	8.4	–13.7^***^	–4.7^***^	–3.8^***^	2018	–8.2^***^
Female	14.2	14.4	13.1	11.1	9.3	9.4	9.0	8.0	6.4	6.3	7.0	6.5	7.6	8.6	7.5	4.8	4.4	5.1	4.5	3.8	–10.4^***^	–6.1^***^	–10.6^***^	2009	–5.3^***^
Middle school	7.3	6.5	6.7	5.0	4.5	4.7	4.4	3.5	2.4	2.2	1.9	1.9	2.6	3.3	3.2	1.9	2.0	2.4	2.3	1.6	–5.7^***^	–6.9^***^	–12.8^***^	2014	–1.0
High school	25.9	23.6	20.3	17.6	14.3	14.2	13.6	12.5	10.2	10.1	11.5	10.4	11.8	13.1	11.4	7.7	7.1	7.9	6.9	6.0	–19.9^***^	–6.2^***^	–13.2^***^	2009	–5.0^***^

1 Using chi-square tests comparing each baseline year (2005, 2015 or 2019) with 2024.

2 The annual percent change (APC) is significantly different from zero.

3 Current cigarette smoking: Proportion of students who smoked cigarettes on ≥1 day during the past 30 days.

4 Current use of electronic vapor products or heated tobacco products: Proportion of students who used electronic vapor products or heated tobacco products on ≥1 day during the past 30 days.

5 Current tobacco product use: Proportion of students who used any tobacco products (cigarettes, heated tobacco products, or electronic vapor products) on ≥1 day during the past 30 days.

6 Current drinking: Proportion of students who consumed at least one glass of alcohol on ≥1 day during the past 30 days.

7 Risky drinking: Proportion of students whose average consumption per occasion during the past 30 days was ≥5 shots of soju for boys, 3 for girls.

*** p<0.001.

**Table 4.** Trends in physical activity and dietary behaviors in the Korea Youth Risk Behavior Survey, 2005–2024

Variables	2005	2006	2007	2008	2009	2010	2011	2012	2013	2014	2015	2016	2017	2018	2019	2020	2021	2022	2023	2024	Difference^1^	APC^2^	APC^2^ and year of any significant change in trend slope		
																					2005/2009/2022 to 2024		Before	Change year	After
Physical activity^3^																									
Male	-	-	-	-	15.7	14.5	15.8	17.3	17.8	19.2	20.5	18.8	19.5	20.3	21.5	19.9	20.7	23.4	24.6	25.1	9.4^***^	3.0^***^	4.6^***^	2014	2.6^***^
Middle school	-	-	-	-	18.0	16.9	18.2	20.4	21.9	22.3	24.0	22.7	23.5	23.9	25.4	22.1	23.6	27.4	29.3	30.8	12.8^***^	3.2^***^	2.6^***^	2021	6.9
High school	-	-	-	-	13.4	12.2	13.4	14.4	13.8	16.2	17.3	15.5	16.2	17.1	18.0	17.8	17.7	19.2	19.8	19.4	6.0^***^	2.8^***^	4.0^***^	2015	2.3^***^
Female	-	-	-		5.4	4.9	5.2	6.1	6.9	8.0	7.4	7.0	7.5	7.1	7.3	7.7	8.1	8.8	9.2	8.9	3.5^***^	3.9^***^	9.0^***^	2014	2.2^***^
Middle school	-	-	-		6.5	5.5	5.7	7.2	8.3	9.6	8.9	9.0	9.4	9.1	8.9	9.6	10.1	11.6	11.6	11.7	5.2^***^	4.1^***^	9.3^***^	2014	2.7^***^
High school	-	-	-		4.4	4.2	4.7	5.0	5.5	6.4	6.0	5.3	5.9	5.3	5.8	5.9	6.0	5.8	6.6	6.0	1.6^***^	2.1^***^	6.6^***^	2014	0.5
Vigorous-intensity physical activity^4^																									
Male	-	-	-	-	-	-	-	-	-	-	-	-	-	-	-	-	-	47.3	52.1	53.5	6.2^***^	-^5^	-	-	-
Middle school	-	-	-	-	-	-	-	-	-	-	-	-	-	-	-	-	-	55.2	61.5	62.5	7.3^***^	-	-	-	-
High school	-	-	-	-	-	-	-	-	-	-	-	-	-	-	-	-	-	38.8	42.4	44.3	5.5^***^	-	-	-	-
Female	-	-	-	-	-	-	-	-	-	-	-	-	-	-	-	-	-	26.3	29.6	28.8	2.5^***^	-	-	-	-
Middle school	-	-	-	-	-	-	-	-	-	-	-	-	-	-	-	-	-	36.6	40.3	40.0	3.4^***^	-	-	-	-
High school	-	-	-	-	-	-	-	-	-	-	-	-	-	-	-	-	-	15.3	18.4	17.3	2.0^***^	-	-	-	-
Skipping breakfast (≥5 day/wk)^6^																									
Male	26.4	25.8	26.2	25.1	28.2	25.5	25.3	24.9	26.7	28.2	26.9	27.3	30.1	32.2	34.6	35.5	37.0	37.4	39.7	40.2	13.8^***^	2.8^***^	–0.9	2012	4.2^***^
Middle school	23.9	22.0	22.7	21.7	26.5	24.3	23.6	24.2	25.7	27.5	26.6	26.7	29.6	31.8	33.4	33.4	35.7	35.6	37.0	37.7	13.8^***^	3.1^***^	1.0	2012	3.9^***^
High school	30.6	30.2	30.2	28.6	29.9	26.7	26.8	25.5	27.7	28.7	27.3	27.8	30.5	32.5	35.8	37.4	38.4	39.4	42.4	42.7	12.1^***^	2.4^***^	–2.2^***^	2013	4.9^***^
Female	28.0	27.7	28.3	26.7	25.9	25.6	23.4	24.6	26.2	28.9	28.9	29.3	33.0	35.1	36.9	39.2	39.1	40.7	42.6	44.7	16.7^***^	3.3^***^	–2.8^***^	2011	5.0^***^
Middle school	25.7	24.5	25.2	24.0	24.2	24.2	22.8	24.3	26.1	28.6	28.4	29.1	32.3	33.4	36.0	37.7	36.5	38.3	40.8	42.9	17.2^***^	3.5^***^	–1.0	2011	4.7^***^
High school	32.0	31.4	31.8	29.5	27.6	27.0	24.0	24.9	26.2	29.2	29.3	29.4	33.6	36.5	37.7	40.7	41.8	43.4	44.5	46.6	14.6^***^	3.2^***^	–5.1^***^	2011	5.3^***^
Fruit consumption (≥1 time/day)^7^																									
Male	32.0	31.0	29.3	33.2	24.0	22.2	19.2	17.4	18.8	20.8	22.5	22.9	21.6	20.8	20.3	19.1	18.4	17.5	16.3	18.3	–13.7^***^	–3.0^***^	–7.8^***^	2011	–1.1
Middle school	36.7	36.3	34.5	38.5	27.8	26.5	22.7	20.4	22.1	24.7	26.8	27.9	26.2	24.5	24.2	22.0	21.9	20.6	19.6	21.5	–15.2^***^	–3.0^***^	–7.6^***^	2011	–1.2
High school	23.8	24.7	23.4	27.6	20.2	18.0	15.8	14.5	15.6	17.2	18.8	18.7	17.8	17.6	16.8	16.3	14.8	14.2	13.0	15.1	–8.7^***^	–2.8^***^	–6.7^***^	2011	–1.5
Female	33.4	33.8	31.0	36.2	25.5	23.7	21.5	20.1	20.8	23.4	23.3	23.5	22.9	20.9	20.6	18.1	17.8	17.0	15.7	19.0	–14.4^***^	–3.6^***^	–7.4^***^	2011	–2.1^***^
Middle school	37.2	38.7	35.6	41.5	30.1	28.6	24.8	23.0	24.3	27.3	27.5	27.7	27.2	25.0	23.8	21.1	20.4	19.2	17.6	21.7	–15.5^***^	–3.6^***^	–6.4^***^	2011	–2.5^***^
High school	26.9	28.0	25.9	30.7	20.9	18.7	18.3	17.3	17.4	19.6	19.5	20.1	19.4	17.3	17.6	15.2	14.9	14.6	13.7	16.2	–10.7^***^	–3.2^***^	–7.1^***^	2011	–1.9^***^

1 p-values were obtained using chi-square tests comparing each baseline year (2005, 2009 or 2022) with 2024.

2 The annual percent change (APC) is significantly different from zero.

3 Physical activity (≥60 min/day, ≥5 day/wk): Proportion of students who engaged in physical activity for at least 60 min/day on ≥5 day/wk.

4 Vigorous-intensity physical activity (≥3 day/wk): Proportion of students who engaged in vigorous-intensity physical activity for at least 3 day/wk.

5 In trend analysis, at least five time points are required to ensure stable trend estimation and statistical validity.

6 Skipping breakfast (≥5 day/wk): Proportion of students who skipped breakfast on ≥5 days during the past 7 days.

7 Fruit consumption (≥1 time/day): Proportion of students who consumed fruit (excluding fruit juice) at least once per day during the past 7 days.

*** p<0.001.

**Table 5.** Trends in mental health in the Korea Youth Risk Behavior Survey, 2005–2024

Variables	2005	2006	2007	2008	2009	2010	2011	2012	2013	2014	2015	2016	2017	2018	2019	2020	2021	2022	2023	2024	Difference^1^	APC^2^	APC^2^ and year of any significant change in trend slope		
																					2005/2020 to 2024		Before	Change year	After
Depressive symptoms^3^																									
Male	25.6	37.1	36.5	34.0	32.1	32.7	28.0	25.5	25.2	22.2	19.7	20.9	20.3	21.1	22.2	20.1	22.4	24.2	21.4	23.1	–2.5^***^	–3.0^***^	–5.3^***^	2016	1.3
Middle school	23.9	33.7	33.4	30.7	29.3	30.0	25.1	23.1	22.9	19.4	16.9	18.2	18.5	19.0	20.1	17.8	21.7	23.1	21.5	22.8	–1.1	–2.9^***^	–6.0^***^	2016	2.9
High school	28.6	41.2	40.1	37.5	35.0	35.4	30.8	27.7	27.4	24.9	22.1	23.1	21.7	22.9	24.1	22.2	23.1	25.3	21.4	23.4	–5.2^***^	–3.1^***^	–4.6^***^	2017	0.4
Female	34.7	46.2	46.6	44.3	43.5	42.6	38.2	36.0	37.1	31.6	27.8	30.5	30.3	33.6	34.6	30.7	31.4	33.5	30.9	32.5	–2.2^***^	–2.1^***^	–3.7^***^	2016	0.5
Middle school	33.3	42.6	43.8	40.4	40.3	39.3	35.4	33.8	35.7	29.9	26.0	27.7	28.9	31.9	34.1	28.4	30.4	33.5	31.5	33.2	–0.1	–1.8^***^	–3.6^***^	2016	1.5
High school	37.1	50.5	49.8	48.5	46.8	45.9	40.9	38.2	38.4	33.1	29.4	32.9	31.6	35.0	35.1	33.0	32.5	33.6	30.2	31.8	–5.3^***^	–2.7^***^	–4.3^***^	2015	–0.5
Generalized anxiety disorder^4^																									
Male	-	-	-	-	-	-	-	-	-	-	-	-	-	-	-	8.0	9.3	9.7	9.0	10.3	2.3^***^	4.6^***^	7.7^***^	2022	2.2^***^
Middle school	-	-	-	-	-	-	-	-	-	-	-	-	-	-	-	6.6	8.8	9.1	8.7	9.6	3.0^***^	8.1^***^	15.6^***^	2022	1.0^***^
High school	-	-	-	-	-	-	-	-	-	-	-	-	-	-	-	9.4	9.8	10.4	9.3	11.1	1.7^***^	3.2	2.9^***^	2022	3.4^***^
Female	-	-	-	-	-	-	-	-	-	-	-	-	-	-	-	14.7	15.6	15.9	16.5	18.0	3.3^***^	4.8^***^	3.4^***^	2022	6.1^***^
Middle school	-	-	-	-	-	-	-	-	-	-	-	-	-	-	-	13.9	15.2	15.9	17.5	18.3	4.4^***^	7.0^***^	7.3^***^	2022	6.8^***^
High school	-	-	-	-	-	-	-	-	-	-	-	-	-	-	-	15.5	16.1	15.9	15.4	17.7	2.2^***^	2.5	–0.3^***^	2022	5.1^***^
Loneliness^5^																									
Male	-	-	-	-	-	-	-	-	-	-	-	-	-	-	-	10.5	12.3	13.9	13.6	14.3	3.8^***^	6.9^***^	13.7^***^	2022	1.4^***^
Middle school	-	-	-	-	-	-	-	-	-	-	-	-	-	-	-	8.2	10.4	12.0	12.1	12.5	4.3^***^	10.6^***^	20.6^***^	2022	1.4^***^
High school	-	-	-	-	-	-	-	-	-	-	-	-	-	-	-	12.6	14.2	15.9	15.3	16.2	3.6^***^	5.8^***^	11.4^***^	2022	1.2^***^
Female	-	-	-	-	-	-	-	-	-	-	-	-	-	-	-	18.0	19.9	21.6	22.9	23.6	5.6^***^	6.8^***^	9.8^***^	2022	4.4^***^
Middle school	-	-	-	-	-	-	-	-	-	-	-	-	-	-	-	17.7	20.0	22.5	24.8	25.2	7.5^***^	9.7^***^	13.6^***^	2022	6.1^***^
High school	-	-	-	-	-	-	-	-	-	-	-	-	-	-	-	18.4	19.9	20.6	20.8	22.0	3.6^***^	4.0^***^	5.2^***^	2022	3.0^***^

1 p-values were obtained using chi-square tests comparing each baseline year (2005 or 2020) with 2024.

2 The annual percent change (APC) is significantly different from zero.

3 Depressive symptoms: Proportion of students who experienced persistent sadness or hopelessness for ≥2 weeks during the past 12 months.

4 Generalized anxiety disorder: Proportion of students scoring ≥10 on the Generalized Anxiety Disorder 7-item scale during the past two weeks.

5 Loneliness: Proportion of students who felt often or always lonely during the past 12 months.

*** p< 0.001.
